# A phase 2 study of AZD4635 in combination with durvalumab or oleclumab in patients with metastatic castration-resistant prostate cancer

**DOI:** 10.1007/s00262-024-03640-6

**Published:** 2024-03-02

**Authors:** Gerald S. Falchook, James Reeves, Sunil Gandhi, David R. Spigel, Edward Arrowsmith, Daniel J. George, Janet Karlix, Gayle Pouliot, Maureen M. Hattersley, Eric T. Gangl, Gareth D. James, Jeff Thompson, Deanna L. Russell, Bhavickumar Patel, Rakesh Kumar, Emerson Lim

**Affiliations:** 1 Drug Development Unit, Sarah Cannon Research Institute at HealthONE, Denver, CO USA; 2 Florida Cancer Specialists South, Sarah Cannon Research Institute, Fort Meyers, FL USA; 3 Florida Cancer Specialists South, Sarah Cannon Research Institute, St. Petersberg, FL USA; 4 Tennessee Oncology, Sarah Cannon Research Institute, Nashville, TN USA; 5 Duke Cancer Institute, Durham, NC USA; 6 Sarah Cannon Research Institute, Gainesville, FL USA; 7 Oncology R&D, AstraZeneca, Waltham, MA USA; 8 BioPharma R&D, AstraZeneca, Boston, MA USA; 9 Medical Statistics Consultancy Ltd, London, UK; 10 Oncology R&D, AstraZeneca, Cambridge, UK; 11 Oncology R&D, AstraZeneca, Gaithersburg, MD USA; 12 Medical Oncology & Hematology-LHCP, Corewell Health Medical Group, Grand Rapids, MI USA

**Keywords:** A_2A_R antagonist, AZD4635, Metastatic castration-resistant prostate cancer, Durvalumab, Oleclumab, Adenosine

## Abstract

**Background:**

Inhibition of the adenosine 2A receptor (A_2A_R) diminishes the immunosuppressive effects of adenosine and may complement immune-targeting drugs. This phase 2 study evaluated the A_2A_R antagonist AZD4635 in combination with durvalumab or oleclumab in patients with metastatic castration-resistant prostate cancer.

**Methods:**

Patients with histologically/cytologically confirmed disease progressing within 6 months on ≥ 2 therapy lines were randomly assigned to either Module 1 (AZD4635 + durvalumab) or Module 2 (AZD4635 + oleclumab). Primary endpoints were objective response rate per RECIST v1.1 and prostate-specific antigen (PSA) response rate. Secondary endpoints included radiological progression-free survival (rPFS), overall survival, safety, and pharmacokinetics.

**Results:**

Fifty-nine patients were treated (Module 1, n = 29; Module 2, n = 30). Median number of prior therapies was 4. One confirmed complete response by RECIST (Module 1) and 2 confirmed PSA responses (1 per module) were observed. The most frequent adverse events (AEs) possibly related to AZD4635 were nausea (37.9%), fatigue (20.7%), and decreased appetite (17.2%) in Module 1; nausea (50%), fatigue (30%), and vomiting (23.3%) in Module 2. No dose-limiting toxicities or treatment-related serious AEs were observed. In Module 1, AZD4635 geometric mean trough concentration was 124.9 ng/mL (geometric CV% 69.84; n = 22); exposures were similar in Module 2. In Modules 1 and 2, median (95% CI) rPFS was 2.3 (1.6 –3.8) and 1.5 (1.3– 4.0) months, respectively. Median PFS was 1.7 versus 2.3 months for patients with high versus low blood-based adenosine signature.

**Conclusion:**

In this heavily pretreated population, AZD4635 with durvalumab or oleclumab demonstrated minimal antitumor activity with a manageable safety profile. Clinical Trial.gov identifier: NCT04089553.

**Supplementary Information:**

The online version contains supplementary material available at 10.1007/s00262-024-03640-6.

## Introduction

Prostate cancer is the second most frequently diagnosed cancer and the fifth leading cause of cancer death in men, estimated to result in over 375,000 deaths worldwide in 2020 [1]. For patients requiring systemic therapy, the mainstay of treatment is hormonal therapy, but patients often develop castration-resistant prostate cancer. For those patients who subsequently develop metastatic castration-resistant prostate cancer (mCRPC), the prognosis is poor with a typically short overall survival (OS) [2].

The current standard of care for mCRPC includes taxanes and novel hormonal agents (NHAs; ie, enzalutamide and abiraterone) [3–5]. Patients who experience disease progression on these types of therapies, have limited therapeutic options [6]. Additionally, many prostate cancer treatments, including docetaxel and cabazitaxel, may not be suitable for all patients and most patients develop refractory disease. Radiotherapy is a mainstay of prostate cancer treatment and local ablative radiation therapy has been shown to prolong progression-free survival (PFS) and OS when combined with androgen-receptor inhibitors [7]. In the phase 3 open-label, VISION trial in patients with mCRPC who had previously progressed on NHA and taxane, radioligand therapy ^177^Lu-PSMA-617 plus standard care significantly prolonged the radiological PFS (rPFS) (median, 8.7 versus 3.4 months, *p* < 0.001) and OS (median, 15.3 versus 11.3 months, *p* < 0.001) compared with standard care alone [8]. The incidence of grade ≥ 3 adverse events (AEs) was higher with ^177^Lu-PSMA-617 than without (52.7% versus 38.0%); however, quality of life was not adversely impacted [8]. Although ^177^Lu-PSMA-617 is approved in the US for select patients with mCRPC, there remains a critical unmet need for additional therapies to treat patients with mCRPC.

Inhibition of the adenosine 2A receptor (A_2A_R) reduces the immunosuppressive effects of adenosine and may complement immune-targeting therapies [9]. AZD4635 is an orally bioavailable A_2A_R antagonist that has demonstrated immunomodulatory and antineoplastic activity [10]. In a phase 1 study (NCT02740985) in patients with advanced solid tumors, AZD4635 monotherapy or in combination with durvalumab (human IgG1 kappa antibody that targets programmed death-ligand 1) was well tolerated and objective responses were observed in patients with mCRPC [11]. An adenosine-signaling gene signature was also measured to look for potential correlations with disease prognosis (ie, median PFS) in these patients. In the Phase 1 study, patients with a high blood‐based adenosine signature had a numerically longer median PFS compared with patients with a low adenosine-signature (21 weeks versus 8.7 weeks, respectively) indicating that adenosine signature may assist in optimal patient selection to achieve better antitumor responses with immunotherapies [11, 12]. Another potential mechanism to reduce the immunosuppressive effect of adenosine could be targeting CD73, an ecto-5′-nucleotidase that converts adenosine monophosphate into extracellular adenosine contributing to tumor growth and metastasis [9, 13]. CD73 upregulation has been observed in patients with various cancers including breast, colon, and thyroid cancer and has been reported as an independent prognostic factor for prostate cancer. CD73 expression in the prostate epithelium exerts immunosuppressive effects while CD73 expression in the tumor stroma were associated with longer recurrence-free survival [14, 15]. Anti-CD73 antibodies, such as oleclumab, can block the conversion of adenosine monophosphate to adenosine and decreases the amount of free adenosine [16], further enhancing the antitumor response.

This phase 2 study evaluated the safety and efficacy of AZD4635 in combination with the immune-targeting drugs durvalumab or oleclumab in patients with mCRPC. Module 1 evaluated AZD4635 in combination with durvalumab and Module 2 evaluated AZD4635 in combination with oleclumab to determine whether a dual adenosine pathway blockade may improve clinical response.

## Methods

### Study design and patients

This open-label, randomized, phase 2a modular study assessed the safety, tolerability, and efficacy of AZD4635 in combination with other immune-modulating therapeutic agents in different treatment modules (NCT04089553). Eligible patients had histologically or cytologically confirmed mCRPC and had previously received and progressed on at least 2 lines of approved systemic therapy for mCRPC within 6 months of enrollment, including a second-generation hormonal agent (eg, abiraterone, enzalutamide, apalutamide). Patients could have had bone-only metastatic disease.

The study was conducted in accordance with the protocol and consensus ethical principles derived from international guidelines including the Declaration of Helsinki and Council for International Organizations of Medical Sciences International Ethical Guidelines, applicable ICH Good Clinical Practice guidelines, and applicable laws and regulations (Independent Ethics Committee/Institutional Review Board). All patients provided written informed consent.

### Randomization and treatment

Patients were randomly assigned using an interactive web-response system to either Module 1 (AZD4635 75 mg orally [PO] once daily [QD] + durvalumab 1500 mg intravenously every 4 weeks [Q4W]) or Module 2 (AZD4635 50 mg [first 25 patients] or 75 mg PO QD + oleclumab 1500 mg IV every 2 weeks [Q2W] for 4 doses, then Q4W). All randomly assigned patients were stratified according to bone-only metastasis or measurable soft-tissue metastasis.

### Endpoints

Primary endpoints were objective response rate (ORR) and prostate-specific antigen (PSA) response rate. Secondary endpoints included rPFS, OS, safety, and pharmacokinetics. PFS by adenosine gene expression signature was an exploratory analysis.

### Assessments

#### Efficacy

ORR was defined as the proportion of patients with a confirmed complete response (CR) or a partial response (PR) and was based on a subset of all dosed patients evaluable for response with measurable disease at baseline per Response Evaluation Criteria in Solid Tumors version 1.1 (RECIST v1.1). PSA confirmed response was defined as the proportion of patients with a reduction in the PSA level of ≥ 50% from baseline to the lowest postbaseline PSA results, measured twice, at least 3 weeks apart using the Prostate cancer working group 3 (PCWG3) criteria. PSA progression was defined as the date of the first PSA increase that was both ≥ 25% and ≥ 2 ng/mL above the nadir and was confirmed by a second value ≥ 3 weeks later, including those progressions that occur after 12 weeks.

rPFS was defined as the time interval from the first dose of AZD4635 until the date of objective disease progression or death (by any cause in the absence of progression). Patients who had not progressed at the time of analysis were censored at the time of the last evaluable RECIST assessment or bone scan. Disease progression was deemed to have occurred if at least 1 of the following criteria was met: soft tissue progression defined by RECIST v1.1, bone lesion progression by PCWG3, or death. Patients with PSA progression were permitted to continue treatment until symptomatic or radiographic progression.

Patients were followed up every 3 months until the last dose of study drug for survival. OS was defined as the time from the first dose of AZD4635 until death due to any cause regardless of whether the patient withdrew from study therapy or received another anticancer therapy. Any patient not known to have died at the time of analysis was censored based on the last recorded date on which the patient was known to be alive.

Patients who discontinued treatment before the occurrence of objective radiographic progressive disease (PD), were followed up with PSA samples, radiographic assessments, and bone scans every 3 months from the last date of the last tumor response assessment until either confirmed objective PD, withdrawal of consent, reaching the data cut-off date for the module, or study or module termination. Patients who discontinued treatment without progression and received a subsequent anticancer therapy other than radiotherapy were not included as responders in the ORR (both visits contributing to a response must have been prior to subsequent therapy for the patient to be considered as a responder). All radiological assessments for determination of ORR were reviewed at the site, with retrospective independent central review conducted, if deemed appropriate.

#### PFS by adenosine gene signature

Blood samples for RNA isolation and subsequent gene expression analysis were collected from 52 patients in Modules 1 (n = 25) and 2 (n = 27) prior to treatment initiation (baseline). Gene expression data were generated by nCounter^®^ (Nanostring Technologies, Inc., Seattle, WA) gene expression assays using the PanCancer Immune Profiling panel (NanoString Technologies, Inc., Seattle, WA) and standard protocol as previously described [11]. The adenosine-signaling levels were assessed using a 14-gene-expression signature (*PPARG, CYBB, COL3A1, FOXP3, LAG3, APP, CD81, GPI, PTGS2, CASP1, FOS, MAPK1, MAPK3*, and *CREB1)* that was previously developed by Sidders and colleagues [12]. Signature scores were calculated as the median of normalized, batch-corrected log_2_ gene-expression values across the 14 genes. Subsequently, the median signature score across all 52 patients was used as the cut-off value for assigning patients to groups with high versus low levels of blood-based adenosine signaling.

#### Safety

AEs, including serious AEs (SAEs), were monitored throughout the treatment and 30-day follow-up periods. Causality for safety endpoints was as attributed by the investigator. The severity of AEs used the Common Terminology Criteria for Adverse Events (CTCAE v5.0). Adverse events of special interest (AESIs) for durvalumab were assessed by the investigator and included events with a potential inflammatory or immune-mediated mechanism possibly requiring more frequent monitoring and/or interventions.

#### Pharmacokinetics

For determination of concentrations of AZD4635 in plasma, 2 mL venous blood samples were collected on day 1 of cycles 1, 3, 5, and 7, and analyzed using a validated bioanalytical method.

### Statistics

Each module was summarized separately. Statistical analyses of all study endpoints are descriptive. In each module, approximately 30 PSA-evaluable patients and approximately 20 patients were to have RECIST-measurable disease at baseline. For all patients, the RECIST tumor-response data were used to determine each patient’s visit response (CR, PR, stable disease, disease progression, or not evaluable [NE]).

ORR and PSA response were assessed using a 2-sided 95% confidence interval (CI) for a single proportion with the exact Clopper–Pearson method. An ORR of 25% and a PSA response of 30% were considered as clinically meaningful responses. Best objective response (BOR) was summarized for dosed patients with measurable disease at baseline and separately for dosed patients evaluable for efficacy. The best PSA percentage change from baseline and the percent change in PSA levels were summarised and plotted. A summary of rPFS (number of events, medians, proportion, and 95% CI for patients who were progression-free at 12 months) was estimated using the Kaplan–Meier method. A 2-sided 95% Cl for the median rPFS was produced. Duration of response (DOR) and OS were analysed similarly (patient numbers permitting). Median adenosine signature was calculated from gene signature data in the evaluable for efficacy analysis set. Values greater than the median were assigned to the high group; values less than the median were assigned to the low group.

Relative dose intensity (RDI) was calculated as RDI = 100% * d/D, where d was the actual cumulative dose delivered up to the earlier of progression (or a censoring event) or the actual last day of dosing and D was the intended cumulative dose up to the earlier of progression (or a censoring event) or the actual last day of dosing plus the protocol-defined postdose rest period.

PK concentrations for AZD4635, durvalumab, and oleclumab were analysed descriptively by summarizing data at predose and/or postdose and data were presented as box plots. Individual concentration data were excluded if there was a missed dose, vomiting after dose administration, or prohibited comedication. Plasma concentrations of AZD4635 and its metabolites were summarized by nominal sample time.

AEs and SAEs were summarized by Medical Dictionary for Regulatory Activities (version 24) system organ class and preferred term, and further categorized by CTCAE grade and causal relationship to study medication. AESIs for durvalumab were summarized. Further details of the study populations are provided in the Supplementary Appendix 1.

## Results

### Patients

**Fig. 1:**
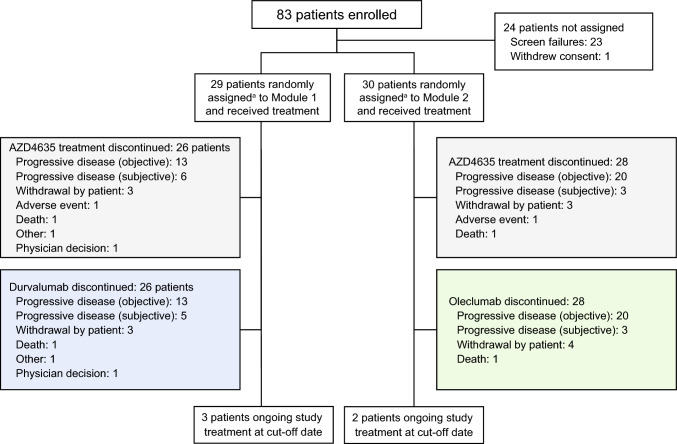
Patient disposition. ^a^If only eligible for 1 module, then a patient was allocated to that module rather than being randomly assigned

As of November 18, 2021, 59 patients had been enrolled and treated at 9 US centers, with 29 patients receiving AZD4635 + durvalumab (Module 1) and 30 receiving AZD4635 + oleclumab (Module 2) (Fig. 1). In Module 1, objective disease progression was the primary reason for AZD4635 treatment discontinuation (13 [45%] patients) and death was the primary reason for study termination (12 [41%] patients). In Module 2, objective disease progression was the primary reason for treatment discontinuation (20 [67%] patients) and completion of treatment was the primary reason for study termination (12 [40%] patients).

Patient demographics and baseline disease characteristics are reported in Table 1 and were consistent with a patient population with mCRPC. Median (range) patient age was 72 (53‒90) years. Most patients were White (80% [47/59]) and had been heavily pretreated (median [range] of 4 [1–9] prior systemic treatments). Overall, 41% (24/59) of patients had > 2 metastatic sites, which were most commonly bone (85% [50/59]), distant lymph nodes (42% [25/59]), and local or regional lymph nodes (42% [25/59]).

**Table 1:** Patient demographics and baseline disease characteristics

Parameter	Module 1 (n = 29)	Module 2 (n = 30)
Median age, years (range)	73 (59 − 90)	72 (53 − 86)
*Race, n (%)*		
White	23 (79.3)	24 (80.0)
Black	5 (17.2)	3 (10.0)
Other	1 (3.4)	3 (10.0)
*ECOG performance status, n (%)*		
0	7 (24.1)	7 (23.3)
1	22 (75.9)	22 (73.3)
2	0	1 (3.3)
Number of prior systemic regimens, median (range)	4 (1 − 9)	4 (2 − 8)
*Number of prior systemic regimens, patient n (%)*		
1	1 (3.4)	0
2	1 (3.4)	4 (13.3)
3	3 (10.3)	9 (30.0)
4	10 (34.5)	6 (20.0)
5	7 (24.1)	6 (20.0)
6	5 (17.2)	2 (6.7)
> 6	2 (6.9)	3 (10.0)
*Type of prior therapy, n (%)*		
Chemotherapy	21 (72.4)	15 (50.0)
Hormonal therapy	27 (93.1)	29 (96.7)
Supportive	12 (41.4)	13 (43.3)
Vaccine	8 (27.6)	10 (33.3)
Other^a^	4 (13.8)	3 (10.0)
*Histology, n (%)*		
Adenocarcinoma	27 (93.1)	29 (96.7)
Carcinoma	1 (3.4)	0
Missing	1 (3.4)	1 (3.3)
*Number of metastatic sites, patient n (%)*		
1	10 (34.5)	11 (36.7)
2	8 (27.6)	6 (20.0)
> 2	11 (37.9)	13 (43.3)
*Metastatic sites, patient n (%)*		
Bone	25 (86.2)	25 (83.3)
Distant lymph nodes	16 (55.2)	9 (30.0)
Liver	7 (24.1)	8 (26.7)
Local or regional lymph nodes	14 (48.3)	11 (36.7)
Lung	3 (10.3)	10 (33.3)
Other	3 (10.3)	7 (23.3)

*ECOG* Eastern cooperative oncology group

^a^Includes investigational agents

### Efficacy

**Fig. 2:**
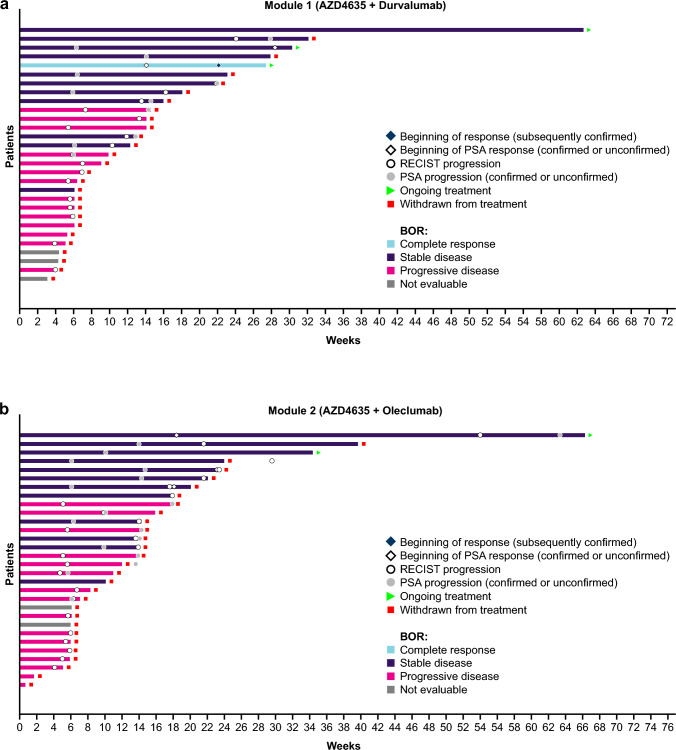
Duration of treatment and best objective response (safety analysis set) for **a** Module 1 and **b** Module 2. *BOR* best objective response, *PSA* prostate-specific antigen, *RECIST* Response Evaluation Criteria in Solid Tumors

Mean (range) total treatment duration for AZD4635 was 3.05 (0.3‒14.4) and 3.22 (0.2‒15.2) months in Modules 1 and 2, respectively (Fig. 2a). An objective response was observed in 1 patient in Module 1 and in no patients in Module 2 (Fig. 2b). Seven (35%) patients in Module 1 and 8 (38%) patients in Module 2 had stable disease for at least 35 days. Ten (50%) patients in Module 1 had progression and 1 (5%) patient died, with the remaining patients having RECIST progression. For Module 2, 11 (52%) patients had progression, 1 (5%) patient died, and the remaining patients had RECIST progression.

**Fig. 3:**
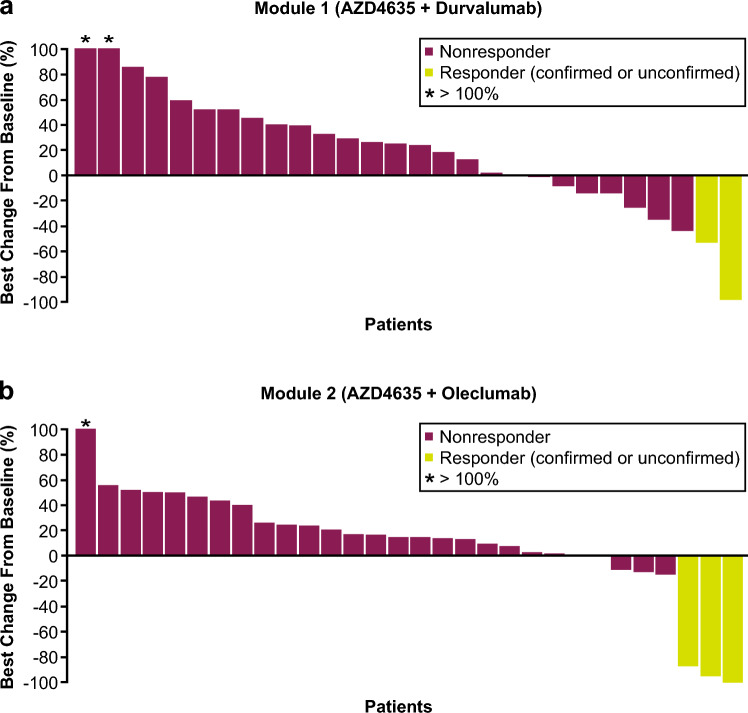
Waterfall plot of best percentage change from baseline in PSA (PSA response analysis set) in **a** Module 1 and **b** Module 2. Best change in PSA is the maximum reduction from baseline or the minimum increase from baseline in the absence of a reduction. For Module 2, the first 25 patients received a starting dose of AZD4635 50 mg. Following a protocol amendment, the starting dose for the remaining patients was 75 mg. Oleclumab was administered IV Q2W for the first 4 doses and Q4W thereafter. *IV* intravenous, *PSA* prostate-specific antigen, *Q2W* every 2 weeks, *Q4W* every 4 weeks

Mean (SD) baseline PSA was 334.2 (593.0) and 176.5 (270.0) ng/mL in Modules 1 and 2, respectively. Five patients had a PSA response (Module 1, n = 2; Module 2, n = 3), with 1 patient each in Modules 1 and 2 having a confirmed PSA response (Fig. 3).

**Fig. 4:**
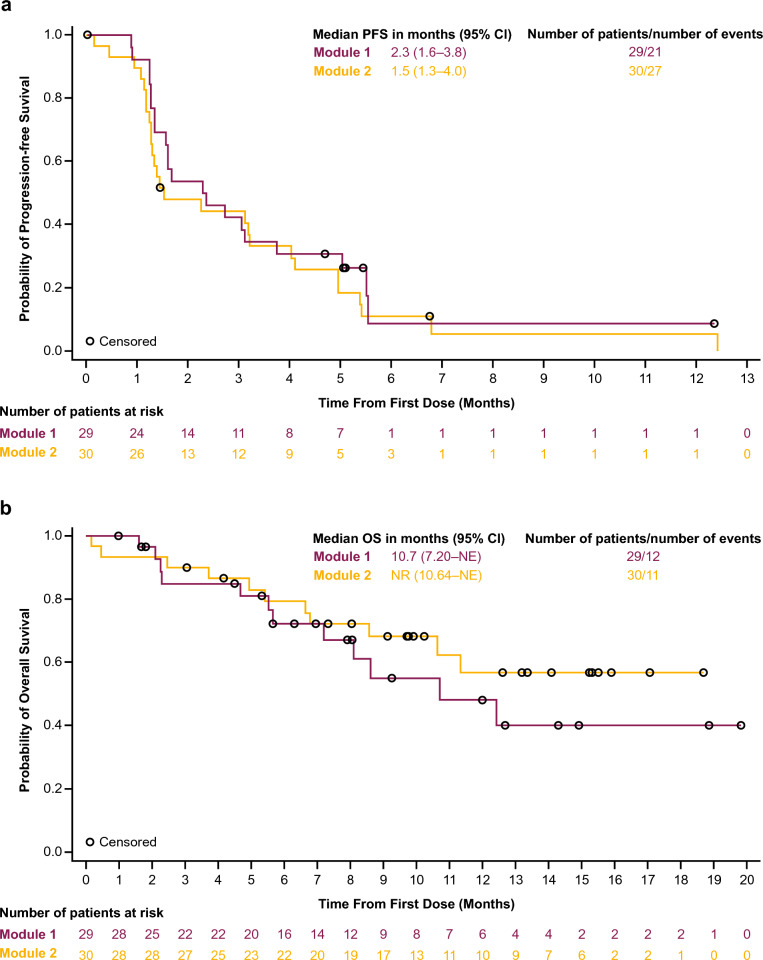
**a** Radiological progression-free survival (efficacy analysis set) and **b** overall survival (safety analysis set). *CI* confidence interval, *NE* not evaluable, *OS* overall survival, *PFS* progression-free survival

Median rPFS was 2.3 (95% CI: 1.6 − 3.8) and 1.5 (95% CI: 1.3 − 4.0) months in Modules 1 and 2, respectively (Fig. 4a). Median OS was 10.7 months (95% CI: 7.2–NE) in Module 1 and not reached in Module 2 (Fig. 4b).

**Fig. 5:**
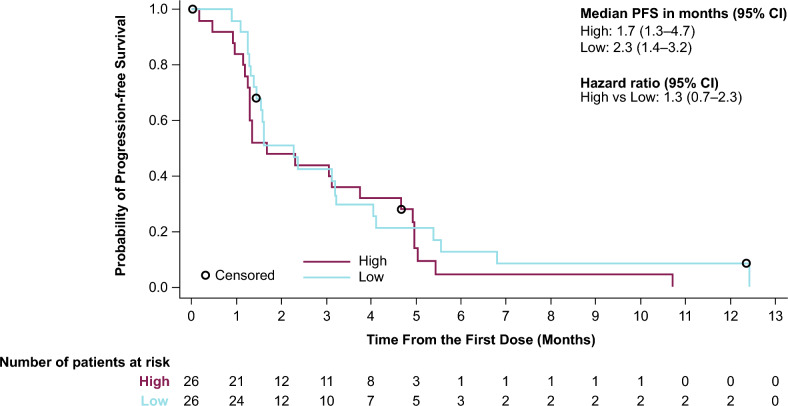
Progression-free survival by median adenosine-signaling gene-signature score (efficacy analysis set). Patients who did not die or have disease progression were censored at their last evaluable response assessment. Values ≥ the median were assigned to the high group; values < the median were assigned to the low group. *CI* confidence interval, *PFS* progression-free survival

In an exploratory analysis of clinical outcome by adenosine-signaling gene signature, no difference was observed in the PFS between patients with relatively high versus low peripheral blood adenosine-signaling levels (1.7 months [95% CI: 1.3–4.7] versus 2.3 months [95% CI: 1.4–3.2], respectively) (Fig. 5).

### Safety

Mean relative dose intensity for AZD4635 was 86.72% and 88.01% in Modules 1 and 2, respectively. Ten (34.5%) patients in Module 1 had dose modifications; 2 of whom had a dose reduction. In Module 2, 15 (50.0%) patients had dose modifications, 5 of whom had a dose reduction. AEs were the most common reason for dose modification (Module 1, n = 7; Module 2, n = 10).

The most frequent AEs possibly related to AZD4635 were nausea (37.9%), fatigue (20.7%), and decreased appetite (17.2%) in Module 1; and nausea (50%), fatigue (30%), and vomiting (23.3%) in Module 2 (Table 2). Three (10.3%) patients in Module 1 and 2 (6.7%) in Module 2 reported at least 1 grade ≥ 3 AE that was possibly related to AZD4635.

**Table 2:** Most common AZD4635-related adverse events (occurring in ≥ 5% of patients in either Module 1 or Module 2)

Preferred term, n (%)^a^	Module 1 (n = 29)	Module 2 (n = 30)	Total (N = 59)
Patients with any possibly related^b^ adverse events	23 (79.3)	25 (83.3)	48 (81.4)
Nausea	11 (37.9)	15 (50.0)	26 (44.1)
Fatigue	6 (20.7)	9 (30.0)	15 (25.4)
Decreased appetite	5 (17.2)	5 (16.7)	10 (16.9)
Vomiting	3 (10.3)	7 (23.3)	10 (16.9)
Diarrhea	4 (13.8)	3 (10.0)	7 (11.9)
Dizziness	4 (13.8)	2 (6.7)	6 (10.2)
Constipation	3 (10.3)	1 (3.3)	4 (6.8)
Insomnia	2 (6.9)	0	2 (3.4)
Hypertension	0	2 (6.7)	2 (3.4)
Thrombocytopenia	0	2 (6.7)	2 (3.4)
Abdominal pain	2 (6.9)	0	2 (3.4)

^a^Patients with events in more than one preferred term are counted once in each of those preferred terms

^b^Possibly related, as assessed by the investigator

No patients had SAEs considered related to AZD4635, durvalumab, or oleclumab. One patient in Module 1 had back pain and muscular weakness and 1 patient in Module 2 had nausea that led to discontinuation of AZD4635. No dose-limiting toxicities or serious treatment-related AEs were observed in either module. There were 3 durvalumab-related AESIs (diarrhea, n = 2; maculopapular rash, n = 1) in Module 1.

In total, there were 23 deaths, 18 of which of which occurred > 28 days after the last dose of study drug. Twelve patients (41.4%) in Module 1 died; 11 of those deaths were considered related to the disease under investigation only. Eleven patients (36.7%) in Module 2 died; 9 of those deaths were considered related to the disease under investigation only. No patients in Module 1 died due to AEs. One patient in Module 2 died due to cardiac arrest, which was considered unrelated to the study treatment.

### Pharmacokinetics

Box plots of concentration of AZD4635 in Module 1 and Module 2 are shown in Supplementary Fig. 1. In Module 1, the geometric mean trough concentration of AZD4635 was 124.9 ng/mL (geometric CV% 69.84; n = 22). Subsequent cycles/days and/or Module 2 results demonstrated similar trough exposures within variability and in some instances with low patient numbers.

## Discussion

Patients with mCRPC who experience disease progression after treatment with taxanes and novel hormonal agents have limited therapeutic options [6]. This phase 2 study evaluated the safety and efficacy of the A_2A_R antagonist AZD4635 in combination with durvalumab or oleclumab in a heavily pretreated population of patients with mCRPC. Limited antitumor activity was observed, with an objective response in 1 patient who received AZD4635 and durvalumab and 5 patients who had a PSA response. AZD4635 combination therapy was generally well tolerated, and no dose-limiting toxicities or serious treatment-related AEs were observed.

In a first-in-human, dose-finding, phase 1 study, patients with relapsed or refractory solid tumors were treated with AZD4635, either alone or in combination with durvalumab [11]. In that study, 8 patients with mCRPC who had received at least 4 prior therapies achieved a confirmed objective response, including 2 who achieved a CR and 4 who achieved a PR with AZD4635 in combination with durvalumab. In patients who received combination therapy, median PFS was 14.9 weeks [11]. These findings supported this phase 2 clinical assessment of AZD4635 combination therapy in patients with mCRPC.

Patients in the phase 1 study treated with AZD4635 alone, or in combination with durvalumab, with a high baseline peripheral adenosine-signaling gene-signature score had numerically longer PFS compared with patients with a low score (medians of 21.0 versus 8.7 weeks, respectively) [11]. In contrast, patients with high baseline peripheral adenosine-signaling gene-signature score in this phase 2 study had a numerically similar median PFS compared with patients with a low score (1.7 months versus 2.3 months, respectively). Although there were no obvious differences between the patient populations in the phase 1 study and the current study that fully explain the disparity in PFS by adenosine-signature outcomes, potential confounding factors (ie, genetics, prior treatment with NHAs or other drugs) could attribute to the differences observed. Further, there was generally less clinical activity, as observed by PSA, ORR, and an overall shorter PFS in the current study compared with the phase 1 study, which may partly explain why the previously reported association between PFS and the baseline adenosine-signaling gene-signature score was not observed here.

Safety findings from this phase 2 study were consistent with the safety data from phase 1 study [11]. In the phase 1 study, an additive effect of durvalumab on the frequency of treatment-related grade ≥ 3 AEs was observed (12.9% with AZD4635 monotherapy versus 21.8% with AZD4635 + durvalumab therapy), which included both immune- and nonimmune-mediated AEs [11]. In this phase 2 study, the frequency of treatment-related grade ≥ 3 AEs was 10.3% in patients who received AZD4635 and durvalumab combination therapy. Although AEs were common in this study, there was only 1 treatment-related AE that led to discontinuation of AZD4635 and there were no SAEs or deaths related to study treatments. Nausea and fatigue are the most common AEs reported with A_2A_R inhibitors [17], but AZD4635 in combination with durvalumab or oleclumab was generally well tolerated. Taken together, there were no new safety signals of AZD4635 in combination with durvalumab and oleclumab that preclude further development of AZD4635 in combination with these therapies.

In addition to the current study, a second phase 2 study (NCT04495179) is investigating the combination of AZD4635 with durvalumab with or without cabazitaxel in patients with mCRPC pretreated with NHA and/or taxanes [18]. These studies were designed following the initial positive outcomes of the phase 1b/2 KEYNOTE-365 trial (NCT02861573) [19]. In the KEYNOTE-365 study, pembrolizumab in combination with docetaxel and prednisone demonstrated antitumor activity with a manageable safety profile in patients with mCRPC who were chemotherapy-naïve and had disease progression on or were intolerant to abiraterone or enzalutamide. The confirmed PSA response rate was 34% with a median rPFS and OS of 8.5 months and 20.2 months, respectively [19]. However, in the phase 3 KEYNOTE-921 trial (NCT03834506), the combination of pembrolizumab and docetaxel did not meet its primary endpoints of an improvement in rPFS and OS [20]. Varied outcomes from these studies highlight the challenges of utilizing checkpoint inhibitors to treat prostate cancer, which is generally referred to as a “cold tumor” due to the lack of T cell infiltration [20, 21]. Myeloid-derived suppressor cells and T regulatory cells present in the tumor microenvironment of cold tumors further dampen the immune response through increased adenosine production via CD39 and CD73 expression [9] Taken together, adenosine blockade may not be sufficient to elicit strong antitumor activity even when combined with checkpoint inhibition. Of note, the minimal antitumor activity observed with AZD4635 in combination with durvalumab or oleclumab is consistent with other checkpoint inhibitors in the mCRPC setting. The results of the phase 2 study of AZD4635 with checkpoint inhibitor and chemotherapy (NCT04495179) will be reported in a separate publication [18].

Strengths of this study include a multicenter design and a representative patient population with heavily pretreated mCRPC. Limitations comprised inherent features of phase 2 studies—including a small patient population, which precluded comparisons between study modules and statistical assessments. Additionally, the follow-up period was relatively short precluding long-term assessment for AEs.

In conclusion, in this population of patients with heavily pretreated mCRPC, treatment with AZD4635 in combination with durvalumab or oleclumab demonstrated minimal antitumor activity with no new safety signals. The lack of efficacy observed in this study is consistent with other checkpoint inhibitors evaluated in the mCRPC setting. These results highlight the limited benefit of checkpoint inhibitor therapy in patients with refractory prostate cancer and a need for novel agents targeting diverse mechanisms.

### Supplementary Information

Below is the link to the electronic supplementary material.

Supplementary file1 (EPS 877 KB)

Supplementary file2 (DOCX 65 KB)

## Data Availability

Data underlying the findings described in this manuscript may be obtained in accordance with AstraZeneca’s data sharing policy described at: https://astrazenecagrouptrials.pharmacm.com/ST/Submission/Disclosure. Data for studies directly listed on Vivli can be requested through Vivli at www.vivli.org. Data for studies not listed on Vivli could be requested through Vivli at https://vivli.org/members/enquiries-about-studies-not-listed-on-the-vivli-platform/. AstraZeneca Vivli member page is also available outlining further details: https://vivli.org/ourmember/astrazeneca/.

## Abbreviations

AE: Adverse event
AESI: Adverse event of special interest
BOR: Best objective response
CI: Confidence interval
CR: Complete response
CTCAE v5.0: Common Terminology Criteria for Adverse Events
DOR: Duration of response
mCRPC: Metastatic castration-resistant prostate cancer
NE: Not evaluable
NHA: Novel hormonal agent
ORR: Objective response rate
OS: Overall survival
PCWG3: Prostate cancer working group 3
PD: Progressive disease
PFS: Progression-free survival
PO: Orally
PR: Partial response
PSA: Prostate-specific antigen
QD: Once daily
Q4W: Every 4 weeks
RECIST v1.1: Response Evaluation Criteria in Solid Tumors version 1.1
RDI: Relative dose intensity
SAE: Serious AEs
