# Supplementary material for: A phase 2 study of AZD4635 in combination with durvalumab or oleclumab in patients with metastatic castration-resistant prostate cancer
Source: Cancer Immunol Immunother. 2024 Mar 2;73(4):72. doi: 10.1007/s00262-024-03640-6 (PMC10908633; doi:10.1007/s00262-024-03640-6)
Supplement: Supplementary file 2 — Supplementary file2 (DOCX 65 KB) [file 262_2024_3640_MOESM2_ESM.docx]

Online-Only Materials / Supplementary Appendix

**Supplementary Figure 1:** **Box plot of concentration of AZD4635 (PK analysis set) in (a) Module 1 and (b) Module 2**


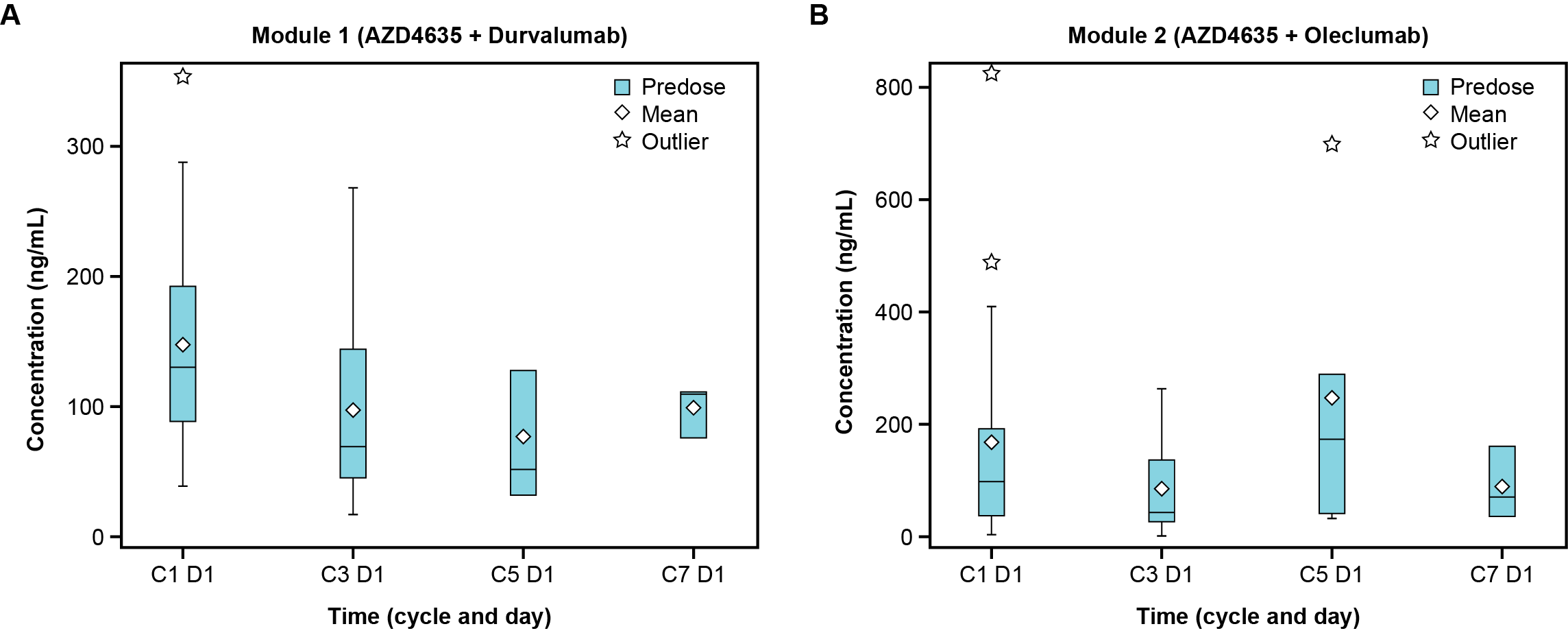


## 1.1 Study populations

| **Population** | **Description** |
| --- | --- |
| Pharmacokinetic | Dosed patients for whom an adequate pharmacokinetic profile had been obtained |
| Tumor response | Dosed patients with a baseline tumor assessment and measurable disease at baseline |
| PSA response | Dosed patients with abnormal baseline PSA data (≥1 ng/mL) |
| Evaluable for efficacy | Dosed patients with a baseline tumor assessment |
| Safety analysis set | All patients who received at least 1 dose of study drug |

PSA, prostate-specific antigen.
